# The deleterious effects of sofosbuvir and ribavirin (antiviral drugs against hepatitis C virus) on different body systems in male albino rats regarding reproductive, hematological, biochemical, hepatic, and renal profiles and histopathological changes

**DOI:** 10.1038/s41598-024-55950-5

**Published:** 2024-03-07

**Authors:** Rana A. Ali, Eatemad A. Awadalla, Yahia A. Amin, Samer S. Fouad, Maha Abd-El Baki Ahmed, Mohammed H. Hassan, Emaad Abdel-Kahaar, Rehab H. Abdel-Aziz

**Affiliations:** 1 Zoology Department, Faculty of Science, South Valley University, Qena, Egypt; 2 Zoology Department, Faculty of Science, Aswan University, Aswan, Egypt; 3 Department of Theriogenology, Faculty of Veterinary Medicine, Aswan University, Aswan, Egypt; 4 Qena University Hospital, South Valley University, Qena, Egypt; 5 Department of Anatomy, Faculty of Medicine, South Valley University, Qena, Egypt; 6 Department of Medical Biochemistry, Faculty of Medicine, South Valley University, Qena, Egypt; 7 Department of Medical Pharmacology, Faculty of Medicine, South Valley University, Qena, Egypt; 8 Institute of Clinical Pharmacology, Medical Faculty Mannheim, Ruprecht-Karls-University, Heidelberg, Germany; 9 Department of Medical Physiology, Faculty of Medicine, South Valley University, Qena, Egypt

**Keywords:** Sofosbuvir, Ribavirin, Hepatitis C virus, Bone marrow suppression, Hypopituitarism, Pancreatitis, Combined immunodeficiency, Cell biology, Chemical biology, Drug discovery, Physiology, Systems biology, Health care, Medical research

## Abstract

Sofosbuvir is one of the crucial drugs used in the treatment of chronic hepatitis C virus (HCV) in adults and children with compensated liver disease, including cirrhosis. It may be used alone or with other drugs. Ribavirin is an antiviral medication used to treat HCV infection. It is not effective when used alone and must be used in combination with other medications, such as sofosbuvir. This study pertains to a comprehensive assessment of the deleterious effects of sofosbuvir (an antiviral drug against chronic HCV) or sofosbuvir combined with ribavirin (an antiviral drug against RNA and DNA viruses) on several biological activities of the body, including hematological, hormonal, biochemical, histological, and immunohistochemical examinations during a long-standing period on male healthy rats. In addition, fertility assessments were performed, including sperm collections and semen parameter investigations. This study was conducted on 21 male rats divided into three equal groups. Group I (control group) received distilled water; group II (sofosbuvir group) received sofosbuvir (4 mg/kg); and group III (sofosbuvir + ribavirin) received sofosbuvir (4 mg/kg) plus ribavirin (30 ml/kg). All groups received the specific drug for six months. Blood and tissue samples were collected for hematological, hormonal, biochemical, histological, and immunohistochemical examinations. In addition, sperm collection and assessments of semen parameters were performed. Results revealed that sofosbuvir causes a highly significant decrease in the mean of most hematological, immunological, hormonal, and biochemical parameters, except for a few numbers of parameters such as neutrophils, monocytes, basophils, cortisol, GOT, and lipase, which exhibit a significant increase. The same occurred in the sofosbuvir + ribavirin group, but at much higher levels, as most hematological, immunological, hormonal, and biochemical parameters exhibit a highly significant decrease except for monocytes, triglyceride, and lipase, which exhibit a significant increase. When compared to the sofosbuvir group alone, the sofosbuvir + ribavirin group demonstrated a highly significant decline in the mean of most hematological, immunological, hormonal, and biochemical parameters except lymphocytes and triglycerides, which exhibit a substantial increase. For the reproductive parameters, both groups exhibit a significant decrease in the total sperm motility percentage. Finally, it can be concluded that sofosbuvir causes acute pancreatitis and combined immunodeficiency. Ribavirin is associated with hormonal deficiency, which indicates the occurrence of hypopituitarism. Moreover, sofosbuvir and ribavirin synergistically affect myelosuppression and cause iron-deficiency anemia. However, sofosbuvir, or its combination with ribavirin, is associated with a reduced risk of hepatocellular carcinoma. Besides, adding ribavirin to be combined with sofosbuvir improved the immunodeficiency caused by sofosbuvir; this confirms that using ribavirin with sofosbuvir reduces the side effects of both alone.

## Introduction

Globally, chronic hepatitis C (CHC) infection affects approximately 56.8 million individuals. Annually, there are approximately 1.5 million new cases reported, resulting in 400,000 fatalities^1^. Egypt is among the nations grappling with a high prevalence of hepatitis C virus (HCV) infection. This condition is linked to the onset of liver cirrhosis, hepatocellular carcinoma, liver failure, and ultimately, mortality, imposing significant economic burdens. Consequently, the elimination of HCV has emerged as a top priority in Egypt’s healthcare agenda^2^. HCV is a single-stranded RNA virus transmitted primarily through blood or blood product exposure.

Treatment of HCV infection with direct-acting antiviral drugs significantly reduces the complications of the disease^3^. In 2011, boceprevir and telaprevir, first-generation protease inhibitors (PIs), along with peg-interferon and ribavirin (PR), were the first direct-acting antivirals (DAAs) that achieved a significant advancement in the treatment of HCV^4–6^. However, this progress was hampered by setbacks from toxicity to resistance^7^. Later, three new DAAs were approved and introduced in several Western countries during the first quarter of 2014. One of the most critical DAAs among these three was sofosbuvir.

Sovaldi (sofosbuvir) is a direct-acting antiviral drug that is approved by the Food and Drug Administration to treat certain types of chronic HCV in adults and children who are at least 3 years old with compensated liver disease, including cirrhosis. It inhibits HCV’s RNA-dependent RNA polymerase NS5B, which is necessary for its replication. The active metabolite of sofosbuvir has no inhibitory effect on human DNA or RNA polymerases or on mitochondrial RNA polymerase, which leads to a favorable safety and side effect profile^8^. A recent study carried out in clinical cases of HCV reported that sofosbuvir-containing regimens have been shown to enhance liver function and reduce viral load in Chinese HCV patients with decompensated cirrhosis^9^. Although antiviral therapy is generally well tolerated, vigilant monitoring of anemia and renal function should be mandatory. Sofosbuvir may be used alone or with other drugs.

Ribavirin (1-β-D-ribofuranosyl-1,2,4-triazole-3-carboxamide) is a purine nucleoside analog with a broad spectrum of activity against a variety of DNA and RNA viral infections^10,11^ and direct antiviral activity against several RNA and DNA viruses. It is used to treat HCV infection. Moreover, it may treat viral hemorrhagic fever and severe acute respiratory syndrome. It is a synthetic guanosine nucleoside and antiviral agent that interferes with the synthesis of viral mRNA^12^. The indirect antiviral properties of ribavirin mediated by the immune system were first observed in treating patients with hepatitis whose symptoms improved without a reduction in the viral load^13^. It is ineffective when used alone and must be combined with other medications, such as sofosbuvir, to treat HCV infection^14^.

The global data on the safety outcomes of sofosbuvir treatment and its combination with ribavirin remained lacking. Furthermore, the drawbacks of sofosbuvir alone, ribavirin alone, or their combination together are unknown. Therefore, the current work aimed to conduct a comprehensive assessment of the deleterious effects of sofosbuvir and sofosbuvir combined with ribavirin on the different biological activities of the body systems, including hematological, hormonal, biochemical, histological, and immunohistochemical examinations during a long-standing period of six months on male albino rats.

## Methodology

### Drugs and chemicals

Sofosbuvir (a product of Mylan Pharmaceuticals, India) is available in tablets with the trade name ‘MyHep.’ Each tablet contains 400 mg of sofosbuvir; these tablets were suspended in distilled water and given to rats orally by gastric tube at 4 mg/kg per day for 6 months^15^.

Ribavirin (a product of Mylan Pharmaceuticals, India) is available in tablets with the trade name ‘VIRACURE.’ Each tablet contains 400 mg of ribavirin; these tablets were suspended in distilled water and given to rats orally by gastric tube at 30 mg/kg per day for 6 months^16^.

### Experimental design

This study included 21 Albino Wistar male rats obtained from the Animal House of the Department of Zoology, Faculty of Science, South Valley University, Qena, Egypt. Their body weights ranged from 150 to 200 g. The rats were housed in wire-mesh cages under a controlled environment (23 + 2 °C, 55% relative humidity, and a 12-h light/dark cycle) and treated according to the guidelines of the Animal House of South Valley University, Faculty of Science, Qena, where standard commercial pellets, which were used as food, and water were provided ad libitum. Other conditions of the animals’ health were maintained during the entire course of the study. Any rats that developed unexpected health issues before or during the experiment were excluded from the study. Table 1 shows exclusion criteria for rats selected for the study^17^. All experimental protocols followed the local institutional guidelines and were approved by the Animal Ethical Committee, South Valley University, Faculty of Science, Qena, Egypt (Code No. 009/03/2023). The included rats were divided randomly into three groups (n = 7 each). All treatments were administered daily via the oral route using intragastric intubation, and rats were treated daily for 6 months as follows:

- Group I (the control group) received distilled water.
- Group II (sofosbuvir group) (Sov): received sofosbuvir (4 mg/kg).
- Group III (sofosbuvir + ribavirin) (Sov + Ri): received sofosbuvir (4 mg/kg) plus ribavirin (30 ml/kg).

**Table 1:** The exclusion criteria for rats selected for the study.

Parameters	Observation
General appearance	Dehydration, decreased body weight, missing anatomy, abnormal posture, hypothermia, fractured appendage, swelling, tissue masses
Skin and fur	Discoloration, urine stain, pallor, redness, cyanosis, icterus, wound, sore, abscess, ulcer, alopecia, ruffled fur
Eyes	Exophthalmos, microphthalmia, ptosis, reddened eye, lacrimation, discharge, opacity
Nose, mouth, and head	Head tilted, nasal discharge, malocclusion, salivation
Respiration	Sneezing, dyspnoea, tachypnoea, rales
Urine	Discolouration, blood in urine, polyuria, anuria
Faeces	Discolouration, blood in the faeces, softness/diarrhoea
Locomotor	Hyperactivity, coma, ataxia, circling, muscle tremors

The study aimed to have a long-term duration of six months to be consistent with the treatment of HCV in humans, which needs a long-term protocol^18^. In addition, part of the treatment protocol included combining sofosbuvir and ribavirin as sofosbuvir in combination with other antiviral agents is approved by the US Food and Drug Administration to treat chronic HCV infection^19^. Recently, it was reported that sofosbuvir plus ribavirin was highly effective for treating patients with CHC genotype 2 and had no serious, treatment-related adverse effects^20^. Furthermore, another study reported that sofosbuvir is considered an add-on therapy to ribavirin for the treatment of chronic hepatitis E in immunocompromised patients^21^.

#### Collection of blood and tissue samples

Rats from various groups were decapitated twenty-four hours after the final dose of therapy. Blood samples were taken from the retro-orbital veins. Blood samples were collected on disodium salt of ethylene diamine tetraacetic acid (EDTA) for hematological analyses (CBC), and the rest of the blood samples were centrifuged at 4000 rpm for 10 min in plain tubes. The cleared serum was separated and kept at  − 80 °C for sex hormones and biochemical analyses. Then, one epididymis was excised from each rat, washed thoroughly using chilled saline (0.9% NaCl), and used for sperm counting. Then, the tissues of the liver, spleen, kidney, and reproductive organs (testicles and epididymis) were excised and fixed in 10% formalin saline. Sections were prepared and stained with different stains for the histological investigations.

#### Hematological examination

The examination of the complete blood picture (platelets count, red blood cell count (RBCs), white blood cell count (WBCs) with differentiation, total hemoglobin, hematocrit assays, and blood indices) was performed by the Automated Hematology Analyzer (Diff3), Mek-6410/MEK-6420.

#### Serum hormone and biochemical assays

Reproductive hormones, including testosterone, follicle-stimulating hormone (FSH), and luteinizing hormone (LH), thyroid hormones such as T_3_, T_4_, and TSH, insulin, and cortisol hormones, were determined using the Intact Immunoassay, a two-site ELISA [Enzyme-Linked Immunosorbent Assay] (ELISA, Labomed, Inc., Los Angeles, USA) reader (EMR-500, Labomed, Inc., Los Angeles, USA) using commercially available ELISA kits (supplied by Chongqing Biospes Co., Ltd., China).

Serum glutamic oxaloacetic transaminase (GOT), serum glutamic pyruvic transaminase (GPT), alkaline phosphatase (ALP) activities, urea, creatinine, uric acid, total triglyceride (mg/dl), cholesterol (mg/dl), and lipase were measured by the colorimetric method using kits from Biodiagnostic commercial kits according to the manufacturer’s instructions.

Alpha-fetoprotein (AFP) was detected by the Human αFP (Alpha-Fetoprotein) ELISA Kit (Cat. No.: E-EL-H0070) using commercially available ELISA kits (supplied by Elabscience®). Adenosine deaminase (*ADA*) was detected by the colorimetric method using the *ADA* Assay Kit, Catalogue No. abx090675, supplied by Abbexa.

#### Sperm collections and assessments of semen parameters

Samples of mature sperm were collected from the caudal region of the epididymis by mincing it finely. Sperm concentration was analyzed using the hemocytometer method. Sperm suspensions from the caudal epididymis were diluted 1:200 with a fixative solution (sodium acid carbonate-formaldehyde solution) and counted according to the procedure indicated in the WHO laboratory manual^22^. The percentage of total sperm motility was determined using a high magnification (400x) light microscope^23,24^.

#### Histological and histochemical examination

Conventional paraffin-embedding techniques have been used to process fixed hepatic and renal tissues. Five μm-thick sections were obtained using microtomes from the prepared paraffin blocks. Subsequently, staining with hematoxylin and eosin was performed on these sections for examination under a light microscope^25^. Furthermore, special stains, including Masson’s trichrome stain, were utilized for additional examination of liver and kidney tissues^26^. Additionally, the periodic acid Schiff’s (PAS) technique was employed to detect collagenous fibers and general carbohydrates^27^, while the mercury-bromophenol blue method, following the recommendation by Mazia et al.^28^, was used for assessing total proteins. The histological and histopathological changes of selected organs were examined under high-power light microscopy (Olympus BX43F, Tokyo 163-0914, Japan).

#### Immunohistochemical examination

Tissue sections (4- to 5-μm thick) were cut from formalin-fixed, paraffin-embedded livers. Sections were deparaffinized and rehydrated by passage through a graded series of ethanol and distilled water. For CD4 immunohistochemistry, the antigen was retrieved by heating the slides in a pressure cooker in tris-buffered saline with 0.075% tween-20 (pH = 7.6) for 10 min. Endogenous peroxidase activity was quenched by incubation with 0.3% v/v H_2_O_2_ in methanol for 20 min at room temperature. Sections were incubated at room temperature for 30 min with a polyclonal mouse anti-human myeloperoxidase antibody (DAKO) diluted at 1:1500. Biotinylated secondary antibodies were used at 1:200 dilution. Immunostaining was performed using an avidin-biotin-horseradish peroxidase system with 3-amino-9-ethylcarbazole as the chromogen. Sections were counterstained with hematoxylin, dehydrated, mounted with DPX, and examined with light microscopy.

#### Statistical analysis

Randomization and blinding techniques were used to allocate and assess the rats to minimize potential biases. All data were analyzed using SPSS version 22.0 (IBM, Armonk, NY, USA). The obtained data were expressed as the mean ± SD and were analyzed using ANOVA (analysis of variance) followed by Tukey’s HSD post hoc test. Statistical significance was considered when *p* ˂ 0.05.

### Ethics approval and consent to participate

The Committee on the Ethics of Animal Experiments of South Valley University’s Faculty of Science approved all the protocols employed. All procedures were followed in compliance with the applicable norms and legislation. The research was conducted following the ARRIVE criteria.

## Results

### Hematological findings

#### Effect of sofosbuvir on Hb, PCV, RBCs, WBCs, platelets, neutrophils, lymphocytes, monocytes, eosinophil, and basophil

When compared with the control group, the sofosbuvir group exhibited a highly significant decrease (*p* < 0.05) in the mean levels of Hb at 11.67 ± 0.57 g/dL compared to 12.43 ± 0.6 g/dL, PCV% at 34.3 ± 2.43% compared to 40.54 ± 1.39%, MCH at 16.82 ± 1.529 pg/cell compared to 21.67 ± 3.652 pg/cell, platelet count at 815.5 ± 40.53 compared to 914.3 ± 61.81, and lymphocytes at 61 ± 3.96% compared to 72.7 ± 1.98%. Additionally, there was a significant increase (*p* < 0.05) in the mean levels of RDW-CV% at 27.48 ± 1.820% compared to 22.22 ± 1.635%, neutrophils at 29.9 ± 2.8% compared to 23.4 ± 1.6%, monocytes at 6.7 ± 3.04% compared to 2.43 ± 0.53%, and basophils at 0.71 ± 0.49% compared to 0.0 ± 0.099%. These findings are summarized in Table 2.

**Table 2:** Changes of the hematological and immunological parameters of control group and other groups treated with oral administration of sofosbuvir and/or its combination with ribavirin in adult male albino rats.

Parameters	Groups		
	G1 (control group)	G2 (Sofosbuvir )	G3 (Sofosbuvir + Ribavirin)
Hb concentration g/dL	12.43 ± 0.6	11.67 ± 0.57^−a^	10.25 ± 0.79^−a−b^
PCV %	40.54 ± 1.39	34.3 ± 2.43^−a^	30.7 ± 2.85^−a−b^
RBCs 10^6^/µl	7.23 ± 0.54	7.61 ± 0.58	6.32 ± 0.87^−a−b^
MCV	51.36 ± 3.6	46.53 ± 2.806	48.53 ± 4.116
MCH pg/cell	21.67 ± 3.6	16.82 ± 1.529^a−^	16.20 ± 0.7642^a−^
RDW-CV%	22.22 ± 1.6	27.48 ± 1.820^a+^	27.83 ± 2.578^a+^
WBCs 10^3^/µl	8.63 ± 0.81	9.29 ± 0.84	7.2 ± 0.48^−a−b^
Platelets 10^3^/µl	914.3 ± 61.81	815.5 ± 40.53^−a^	615.3 ± 64.99^−a−b^
Neutrophil %	23.4 ± 1.6	29.9 ± 2.8^+a^	18.7 ± 2.2^−a−b^
Lymphocytes %	72.7 ± 1.98	61 ± 3.96^−a^	71 ± 2.31^+b^
Monocytes %	2.43 ± 0.53	6.7 ± 3.04^+a^	8.29 ± 1.38^+a^
Eosinophil %	1.43 ± 0.53	1.71 ± 0.49	1.86 ± 0.38
Basophil %	0.0 ± 0.0	0.71 ± 0.49^+a^	0.14 ± 0.38^−b^

The results presented Mean ± S.D. of seven rats.

^a^significantly different from control group at (*p* < 0. 05).

^b^significantly different from G2 (Sofosbuvir group) at (*p* < 0. 05).

#### Effect of sofosbuvir with ribavirin on Hb, PCV, RBCs, WBCs, platelets, neutrophils, lymphocytes, monocytes, eosinophil and basophil:

When compared with the control group, the sofosbuvir with ribavirin group exhibited a highly significant decrease (*p* < 0.05) in the mean levels of Hb at 10.25 ± 0.79 g/dL compared to 12.43 ± 0.6 g/dL, PCV% at 30.7 ± 2.85% compared to 40.54 ± 1.39%, RBCs at 6.32 ± 0.87 million/μL compared to 7.61 ± 0.58 million/μL, MCH at 16.20 ± 0.7642 pg/cell compared to 21.67 ± 3.652 pg/cell, WBCs at 7.2 ± 0.48 thousand/μL compared to 8.63 ± 0.81 thousand/μL, and platelet count at 615.3 ± 64.99 thousand/μL compared to 914.3 ± 61.81 thousand/μL. Additionally, there was a significant increase (*p* < 0.05) in the mean levels of RDW-CV% at 27.83 ± 2.578% compared to 22.22 ± 1.635%, and monocytes at 8.29 ± 1.38% compared to 2.43 ± 0.53%. These findings are summarized in Table 2.

#### Difference between sofosbuvir and sofosbuvir with ribavirin groups in Hb, PCV, RBCs, WBCs, platelets, neutrophil, lymphocytes, monocytes, eosinophil, and basophil

When compared with the sofosbuvir group, the sofosbuvir with ribavirin group displayed a highly significant decrease (*p* < 0.05) in the mean levels of Hb at 10.25 ± 0.79 g/dL compared to 11.67 ± 0.57 g/dL, PCV% at 30.7 ± 2.85% compared to 34.3 ± 2.43%, RBCs at 6.32 ± 0.87 million/μL compared to 7.61 ± 0.58 million/μL, WBCs at 7.2 ± 0.48 thousand/μL compared to 9.29 ± 0.84 thousand/μL, and platelet count at 615.3 ± 64.99 thousand/μL compared to 815.5 ± 40.53 thousand/μL. Furthermore, there was a significant decrease (*p* < 0.05) in the mean levels of neutrophils at 18.7 ± 2.2% compared to 29.9 ± 2.8% and basophils at 0.14 ± 0.38% compared to 0.71 ± 0.49%. Conversely, there was a significant increase (*p* < 0.05) in the mean levels of lymphocytes at 71 ± 2.31% compared to 61 ± 3.96%. These findings are summarized in Table 2.

#### Effect of sofosbuvir and sofosbuvir with ribavirin on sperm concentration, total sperm motility, free testosterone level, FSH, and LH

Compared with the control group, the sofosbuvir group exhibited a highly significant decrease (*p* < 0.05) in the mean total sperm motility percentage; with values of 55.29 ± 7.67% compared to 78.29 ± 4.15%. These findings are presented in Table 3.

**Table 3:** Evaluation of sperm concentration, total sperm motility, free testosterone level, FSH and LH of control group and other groups treated with oral administration of sofosbuvir and/or its combination with ribavirin in adult male albino rats.

	G1 (control group)	G2 (Sofosbuvir)	G3 (Sofosbuvir + Ribavirin)
Sperm concentration (million/ml)	182.86 ± 6.8	181.86 ± 9.84	181.6 ± 10.55
Total sperm motility percent (%)	78.29 ± 4.15	55.29 ± 7.67^−a^	57.43 ± 6.02^−a^
Testosterone (pg/ml)	0.27 ± 0.06	0.25 ± 0.09	0.36 ± 0.08^+b^
FSH (mIU/ml)	0.02 ± 0.01	0.03 ± 0.02	0.03 ± 0.02
LH (mIU/ml)	0.004 ± 0.002	0.003 ± 0.003	0.003 ± 0.001

The results presented as Mean ± S.D. of seven rats.

^a^significantly different from control group at (*p* < 0. 05).

^b^significantly different from G2 (Sofosbuvir group) at (*p* < 0. 05).

Similarly, the sofosbuvir with ribavirin group displayed a highly significant decrease (*p* < 0.05) in the mean total sperm motility percentage when compared with the control group, with values of 57.43 ± 6.02% compared to 78.29 ± 4.15% (Table 3).

Moreover, when compared with the sofosbuvir group, the sofosbuvir with ribavirin group demonstrated a highly significant increase (*p* < 0.05) in the mean testosterone level, with values of 0.36 ± 0.08 pg/ml compared to 0.25 ± 0.09 pg/ml (Table 3).

#### Effect of sofosbuvir and sofosbuvir with ribavirin on T_3_, T_4_, TSH, insulin, cortisol, and AFP

Compared with the control group, the sofosbuvir group exhibited a highly significant decrease (*p* < 0.05) in the mean levels of T_4_ at 0.37 ± 0.08 ng/dl compared to 0.51 ± 0.06 ng/dl, TSH at 0.0012 ± 0.001 uIU/ml compared to 0.007 ± 0.007 uIU/ml, insulin at 72.57 ± 7.35 uIU/ml compared to 93.14 ± 8.55 uIU/ml, and AFP at 0.49 ± 0.08 ng/ml compared to 0.7 ± 0.1 ng/ml. Additionally, there was a significant increase (*p* < 0.05) in the mean cortisol level at 0.55 ± 0.09 ng/ml compared to 0.39 ± 0.09 ng/ml (Table 4).

**Table 4:** Evaluation of thyroid hormones, insulin, cortisol and AFP of control group and other groups treated with oral administration of sofosbuvir and/or its combination with ribavirin in adult male albino rats.

	G1 (control group)	G2 (Sofosbuvir)	G3 (Sofosbuvir + Ribavirin)
T3 (pg/ml)	2.19 ± 0.22	2.36 ± 0.23	2.18 ± 0.08
T4 (ng/dl)	0.51 ± 0.06	0.37 ± 0.08^−a^	0.32 ± 0.09^−a^
TSH (uIU/ml)	0.007 ± 0.007	0.0012 ± 0.001^−a^	0.001 ± 0.001^−a^
Insulin (uIU/ml)	93.14 ± 8.55	72.57 ± 7.35^−a^	77.71 ± 8.24^−a^
Cortisol (ng/ml)	0.39 ± 0.09	0.55 ± 0.09^+a^	0.48 ± 0.11
AFP (ng/ml)	0.7 ± 0.1	0.49 ± 0.08^−a^	0.44 ± 0.12^−a^

The results presented Mean ± S.D. of seven rats.

^a^significantly different from control group at (*p* < 0. 05).

bsignificantly different from G2 (Sofosbuvir group) at (*p* < 0. 05).

Similarly, the sofosbuvir with ribavirin group, when compared with the control group, demonstrated a highly significant decrease (*p* < 0.05) in the mean levels of T_4_ at 0.32 ± 0.09 ng/dl compared to 0.51 ± 0.06 ng/dl, TSH at 0.001 ± 0.001 uIU/ml compared to 0.007 ± 0.007 uIU/ml, insulin at 77.71 ± 8.24 uIU/ml compared to 93.14 ± 8.55 uIU/ml, and AFP at 0.44 ± 0.12 ng/ml compared to 0.7 ± 0.1 ng/ml (Table 4).

No significant difference was observed between the sofosbuvir and sofosbuvir with ribavirin groups regarding T_4_, TSH, insulin, and AFP levels.

#### Effect of sofosbuvir and sofosbuvir with ribavirin on ALP, GOT, GPT, creatinine, urea, uric acid, ADA, triglyceride, cholesterol, and lipase

Compared with the control group, the sofosbuvir group demonstrated a highly significant decrease (*p* < 0.05) in the mean level of *ADA* at 40.86 ± 0.81 U/L compared to 47.02 ± 0.58 U/L. Additionally, there was a significant increase (*p* < 0.05) in the mean levels of GOT at 96.86 ± 4.18 U/L compared to 84.29 ± 10.13 U/L and lipase at 17.71 ± 0.95 U/L compared to 13.0 ± 1.41 U/L. These results are presented in Table 5.

**Table 5:** detection of serobiochemical parameters of control group and other groups treated with oral administration of sofosbuvir and/or its combination with ribavirin in adult male albino rats.

Groups	G1 (control group)	G2 (Sofosbuvir )	G3 (Sofosbuvir + Ribavirin )
ALP (U/L)	270.0 ± 15.77	283.86 ± 29.24	139.29 ± 10.81^−a−b^
GOT (U/L)	84.29 ± 10.13	96.86 ± 4.18^+a^	77.57 ± 14.99^−b^
GPT (U/L)	24.29 ± 4.68	26.57 ± 7.02	17.57 ± 4.31^−a−b^
Creatinine (mg/dl)	0.58 ± 0.07	0.57 ± 0.08	0.61 ± 0.06
Urea (mg/dl)	47.14 ± 2.61	45.29 ± 1.6	45.29 ± 2.36
Uric acid (mg/dl)	1.47 ± 0.21	1.59 ± 0.28	1.46 ± 0.45
Triglyceride (mg/dl)	107.29 ± 14.84	121.7 ± 23.64	143.86 ± 14.42^+a+b^
Cholesterol (mg/dl)	67.28 ± 5.28	69 ± 18.31	70 ± 13.9
Lipase (U/L)	13.0 ± 1.41	17.71 ± 0.95^+a^	15.71 ± 1.79^+a−b^
ADA (U/L)	47.02 ± 0.58	40.86 ± 0.81^−a^	45.57 ± 4.24^+b^

The results presented Mean ± S.D. of seven rats.

^a^significantly different from control group at (*p* < 0. 05).

^b^significantly different from G2 (Sofosbuvir group) at (*p* < 0. 05).

Similarly, the sofosbuvir with ribavirin group, when compared with the control group, exhibited a highly significant decrease (*p* < 0.05) in the mean levels of ALP at 139.29 ± 10.81 U/L compared to 270.0 ± 15.77 U/L, and GPT at 17.57 ± 4.31 U/L compared to 24.29 ± 4.68 U/L. Moreover, there was a significant increase (*p* < 0.05) in the mean levels of triglycerides at 143.86 ± 14.42 mg/dl compared to 107.29 ± 14.84 mg/dl and lipase at 15.71 ± 1.79 U/L compared to 13.0 ± 1.41 U/L (Table 5).

Furthermore, the sofosbuvir with ribavirin group, when compared with the sofosbuvir group, demonstrated a highly significant decrease (*p* < 0.05) in the mean levels of ALP at 139.29 ± 10.81 U/L compared to 283.86 ± 29.24 U/L, GOT at 77.57 ± 14.99 U/L compared to 96.86 ± 4.18 U/L, and GPT at 17.57 ± 4.31 U/L compared to 26.57 ± 7.02 U/L, as well as lipase at 15.71 ± 1.79 U/L compared to 17.71 ± 0.95 U/L. Moreover, there was a significant increase (*p* < 0.05) in the mean levels of *ADA* at 45.57 ± 4.24 U/L compared to 40.86 ± 0.81 U/L and triglycerides at 143.86 ± 14.42 mg/dl compared to 121.7 ± 23.64 mg/dl (Table 5).

### Histopathological results of the organs among study groups

#### Bone marrow

**Figure 1:**
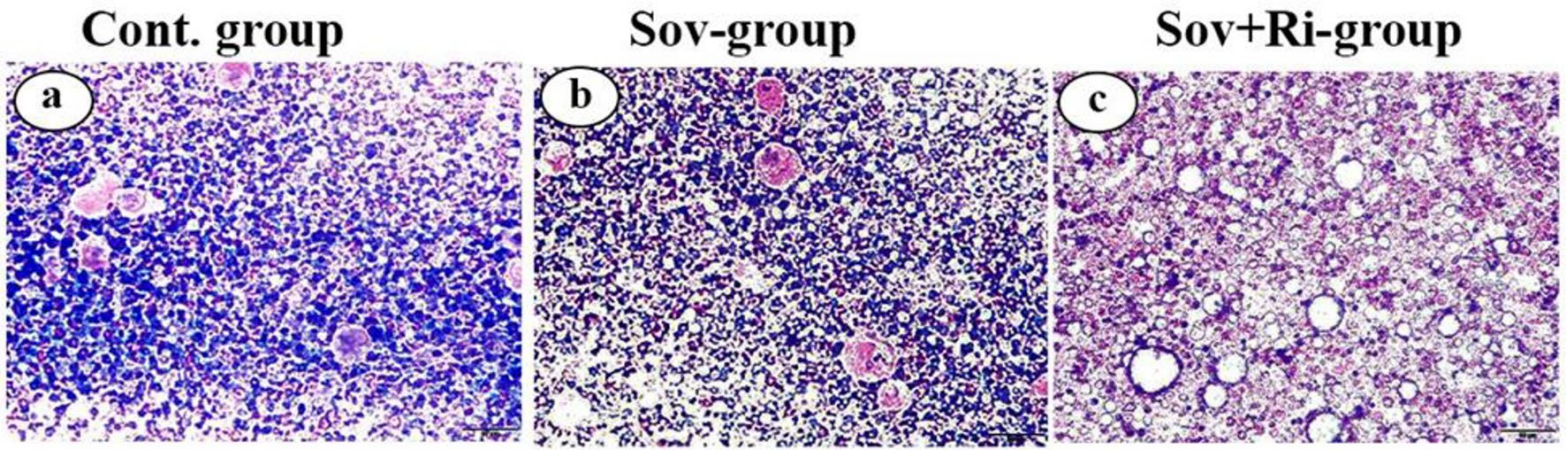
(**a**–**c**): Photomicrographs of male albino rat’s bone marrow stained with Giemsa stain (Scale bar 50 μm for all figures) (**a**): (Cont. group) Photomicrograph of rat bone marrow of control group. (**b**): (Sov-group) Photomicrograph of rat bone marrow of Sofosbuvir treated group. (**c**): (Sov + Ri-group) Photomicrograph of rat bone marrow of Sofosbuvir + ribavirin treated group.

Hematoxylin and eosin (H&E) staining displayed abundant bone marrow cells in the control group (Fig. 1a). Administration of sofosbuvir led to a significant (*p* < 0.05) reduction in the number of bone marrow cells compared to the control group (Fig. 1b). Treatment with a combination of sofosbuvir and ribavirin resulted in a further significant (*p* < 0.05) decrease in the number of bone marrow cells compared to treatment with sofosbuvir alone (Fig. 1c).

#### Spleen

**Figure 2:**
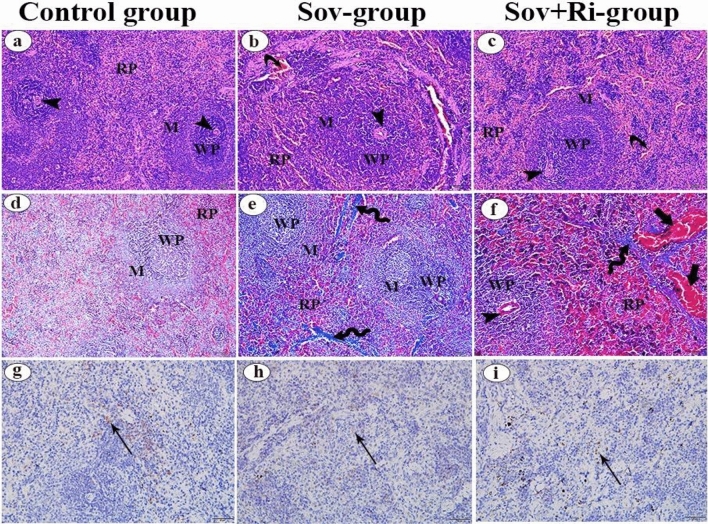
(**a**–**i**): Photomicrographs of male albino rat’s splenic sections (X 200 = 100 μm). (**a**–**c**): Photomicrographs of rat spleen stained with H&E. (**d**–**f**): Photomicrographs of rat spleen stained with Masson’s trichrome stain. (**g**–**i**): Photomicrographs of rat spleen prepared for Cd4 receptors immune-staining. Red pulp (RP), White pulp (WP), Marginal zone (M), Central artery (arrow head), Slightly congestion of the blood sinusoids of the red pulp (), Thickened trabecula with large amount of collagen fibers (), Markedly congested blood vessels (thick arrow), Positive expression of CD4 protein (↑), H&E: haematoxylin and eosin, SOV: Sofosbuvir; Ri: Ribavirin.

Examination of hematoxylin and eosin-stained splenic sections from both treated groups (sofosbuvir and sofosbuvir in combination with ribavirin groups) (Fig. 2b and c, respectively) revealed histological similarities with each other and with the control group (Fig. 2a). All group sections showed distinct splenic follicles with normal white and red pulp. The red pulp consisted of blood sinusoids and splenic cords, while the white pulp comprised aggregations of lymphocytes surrounding the central arterioles, with the periarteriolar lymphatic sheath and marginal zone being observed as components of the white pulp. However, the blood sinusoids were slightly congested in the red pulp throughout the splenic sections of both treated groups.

Examination of Masson’s trichrome-stained splenic sections from the control group revealed thin trabeculae with few collagen fibers (Fig. 2d). However, in the trabeculae traversing the central part of the splenic section of the sofosbuvir group (Fig. 2e), there was an increased amount of collagen fibers compared to the control group. Moreover, markedly thickened trabeculae with an increased amount of collagen fibers and markedly congested blood vessels were observed in the spleen of the sofosbuvir with ribavirin group (Fig. 2f) compared to both the control group and the sofosbuvir group.

Anti-CD4 protein immunostained spleen sections showed few cells with a positive reaction in all studied groups, while other cells showed a negative reaction (Fig. 2g,h, and i).

#### Testis

**Figure 3:**
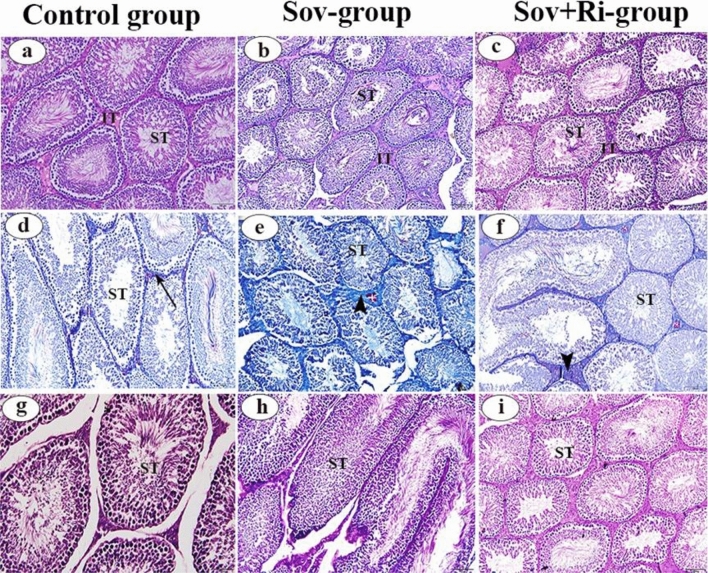
(**a**–**i**): Photomicrographs of male albino rat’s testicular sections (X 200 = 100 μm). (**a**–**c**): Photomicrographs of rat testis stained with H&E. (**d**–**f**): Photomicrographs of rat testis stained with Masson’s trichrome stain. (**g**–**i**): Photomicrographs of rat testis stained with PAS reaction. Seminiferous tubules (ST), interstitial connective tissue (IT), distribution of collagenous fibers (arrow), A tiny increase in the amount of collagenous fibres (arrow head), H&E: haematoxylin and eosin; PAS: Periodic acid–Schiff; SOV: Sofosbuvir; Ri: Ribavirin.

Examination of hematoxylin and eosin-stained testicular sections from the Sov and Sov + Ri groups (Fig. 3b and c, respectively) showed histological similarities with each other and with the control group (Fig. 3a). The testicular examination across all group sections revealed a healthy histological structure characterized by well-developed seminiferous tubules and complete spermatogenesis.

Examination of Masson’s trichrome-stained sections revealed a moderate increase in the amount of connective tissue in the testes of both the Sov and Sov + Ri groups (Fig. 3e and f, respectively) compared to the control group (Fig. 3d), which displayed a normal appearance of interstitial connective tissue.

Examination of the testicular sections from the Sov and Sov + Ri groups (Fig. 3h and i, respectively) revealed a strong, intense PAS-positive reaction in the basement membranes surrounding seminiferous tubules, spermatogenic epithelium, and interstitial connective tissue, akin to the appearance observed in the control testes section (Fig. 3g).

#### Epididymis

**Figure 4:**
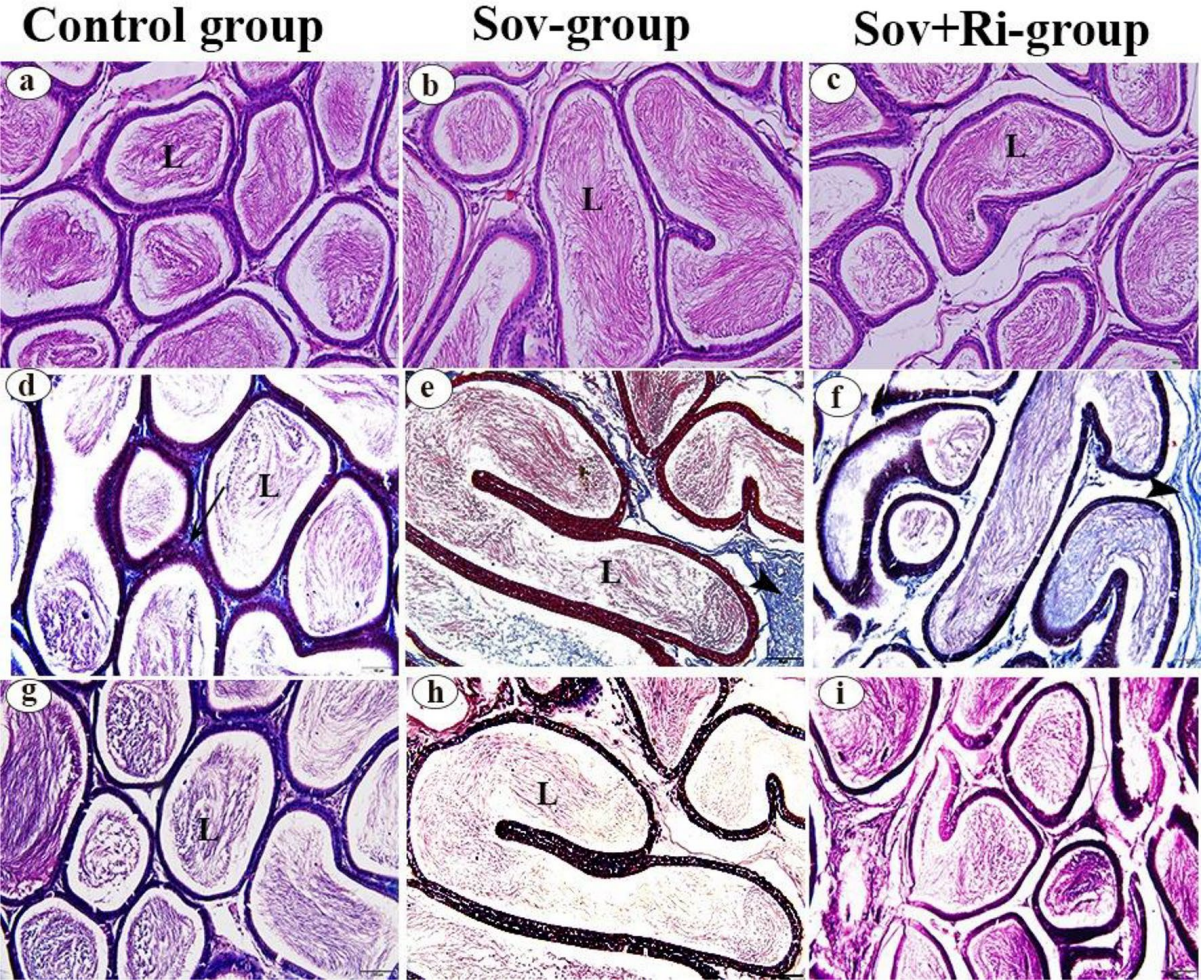
(**a**–**i**): Photomicrographs of epididymal sections of male albino rats (X 200 = 100 μm). (**a**–**c**): Rat epididymal sections stained with H&E. (**d**–**f**): Rat epididymal sections stained with Masson’s trichrome stain. (**g**–**i**): Rat epididymal sections stained with PAS reaction. Epididymal ducts (L), Normal distribution of collagenous fibers (arrows), Marked increase in the amount of collagenous fibers (arrow head), H&E: haematoxylin and eosin; PAS: Periodic acid–Schiff; SOV: Sofosbuvir; Ri: Ribavirin.

Examination of hematoxylin and eosin-stained epididymal sections from the Sov and Sov + Ri groups (Fig. 4b and c, respectively) revealed histological similarities with each other and with the control group (Fig. 4a). The examination across all groups’ sections demonstrated the normal structure of epididymal ducts with normal sperm density, consistent with observations in the control group.

Masson’s trichrome-stained epididymal sections from the Sov and Sov + Ri groups (Fig. 4e and f, respectively) displayed an increase in the amount of collagen fibers in the interstitial connective tissue surrounding epididymal ducts compared to the control group (Fig. 4d), which exhibited a normal distribution of collagen fibers around epididymal ducts.

PAS staining of the epididymal sections from the Sov and Sov + Ri groups (Fig. 4h and i) revealed strong, intense PAS-positive material in the lumina of the epididymal ducts, consistent with observations in the control group (Fig. 4g).

#### Liver

**Figure 5:**
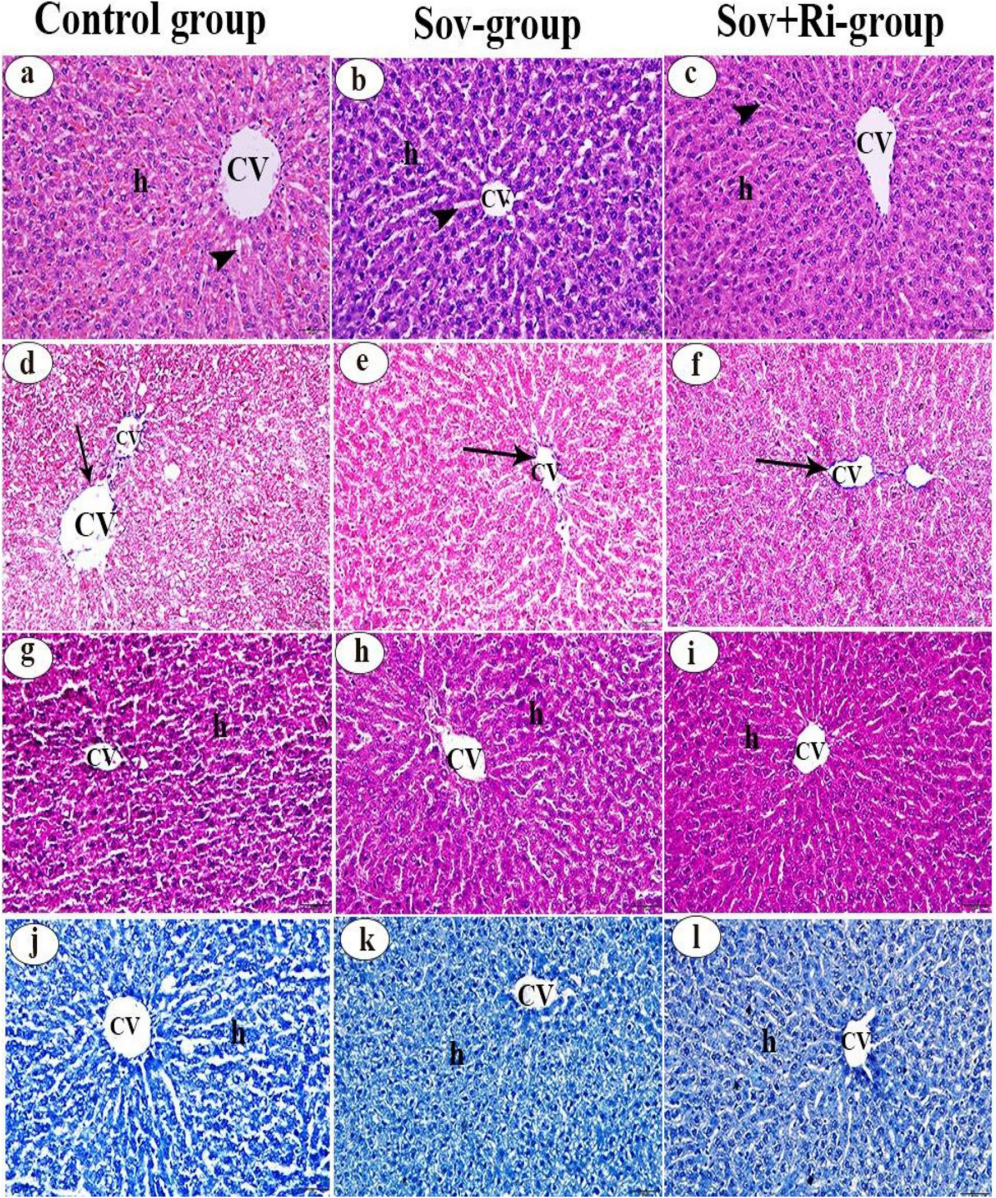
(**a**–**l**): Photomicrographs of male albino rat’s liver sections (X 400 = 50 µm). (**a**–**c**): Photomicrographs of rat liver stained with H&E. (**d**–**f**): Photomicrographs of rat liver stained with Masson’s trichrome stain. (**g**–**i**): Photomicrographs of rat liver stained with PAS reaction. (**j**–**l**): Photomicrographs of rat liver stained with bromophenol blue stain. Hepatocytes (h), Central vein (CV), Blood sinusoids (arrow head), Normal distribution of collagenous fibres (arrow). H&E: haematoxylin and eosin; PAS: Periodic acid–Schiff; SOV: Sofosbuvir; Ri: Ribavirin.

Examination of hematoxylin and eosin-stained hepatic sections from the Sov and Sov + Ri groups (Fig. 5b and c) displayed a healthy pattern similar to that observed in the control group (Fig. 5a). In the control rats, the liver appeared as a continuous mass of hepatocytes with a cord-like pattern and a relatively large number of blood sinusoids. The hepatocytes exhibited polygonal shapes with centrally located nuclei and homogeneous eosinophilic cytoplasm around the central vein. Some red blood corpuscles were present in the blood sinusoids.

Examination of Masson’s trichrome stain under light microscopy revealed a small amount of collagenous connective tissue fibers around the central veins and sinusoids (normal distribution) in the liver of the control group and all experimental groups, Sov and Sov + Ri groups (Fig. 5d,e, and f, respectively).

Histochemically, utilization of the PAS technique presented a distinct distribution of polysaccharide materials in the hepatocytes of the control group and all experimental groups, Sov and Sov + Ri groups (Fig. 5g,h, and i, respectively). The hepatocyte cytoplasm exhibited magenta granules, indicating the presence of carbohydrates.

Furthermore, sections stained with mercury bromophenol blue from the Sov and Sov + Ri groups (Fig. 5k and l, respectively) revealed similar findings to those in the control group (Fig. 5j). The hepatocyte nucleus and cell membrane were intensely blue-stained. In contrast, the cytoplasm was moderately blue-stained with mercuric bromophenol blue, indicating the presence of proteins.

#### Kidney

**Figure 6:**
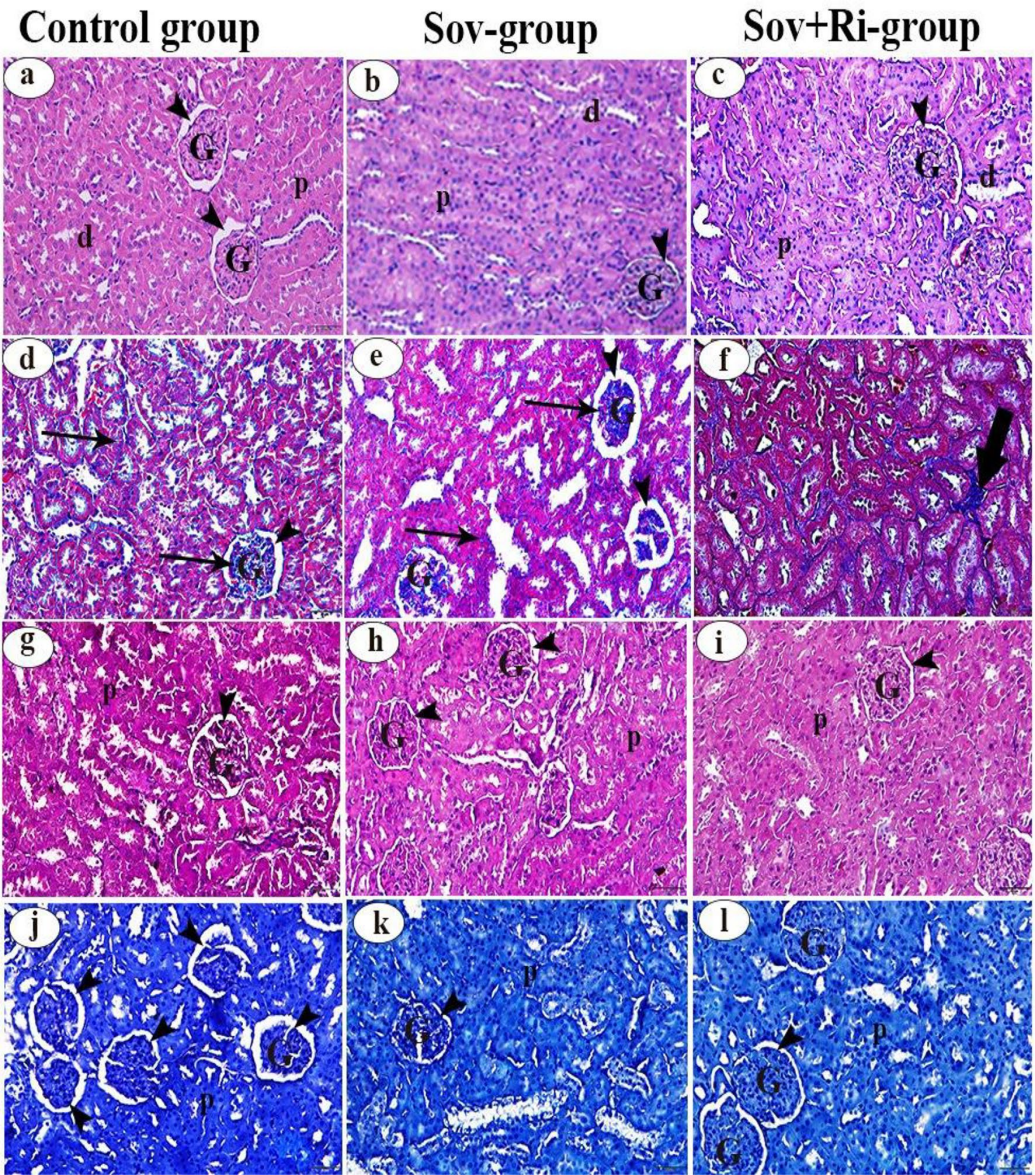
(**a**–**l**): Photomicrographs of male albino rat’s kidney sections (X 400 = 50 µm). (**a**–**c**): Photomicrographs of rat kidney stained with H&E. (**d**–**f**): Photomicrographs of rat kidney stained with Masson’s trichrome stain. (**g**–**i**): Photomicrographs of rat kidney stained with PAS reaction. (**j**–**l**): Photomicrographs of rat kidney stained with bromophenol blue stain. Glomerulus (G), Bowman’s space (arrow head), Proximal convoluted tubule (p), Diatal convoluted tubules (d), Normal distribution of collagenous fibres (arrow), An increase in the amount of collagenous fibres (thick arrow). H&E: haematoxylin and eosin; PAS: Periodic acid–Schiff; SOV: Sofosbuvir; Ri: Ribavirin.

Examination of hematoxylin and eosin-stained renal sections from both the Sov and Sov + Ri groups (Fig. 6b and c, respectively) revealed no apparent lesions. It displayed a normal histological architecture similar to the control group (Fig. 6a). The renal corpuscles exhibited tufts of glomeruli and Bowman’s space. Proximal convoluted tubules showed narrow lumina lined with high-cuboidal cells having rounded basal nuclei and deeply acidophilic cytoplasm, while distal convoluted tubules showed wider lumina lined with cubical cells having rounded central nuclei and paler acidophilic cytoplasm.

Examination of Masson’s trichrome-stained renal sections from the sofosbuvir group (Fig. 6e) exhibited a similar pattern to the control group (Fig. 6d), displaying fine blue-colored stripes (collagen fibers) among glomerular capillaries and between renal tubules. However, there was mild deposition of collagen fibers between the renal tubules in the sofosbuvir with the ribavirin group (Fig. 6f).

The PAS reactivity displayed an intense magenta color in the control renal sections (Fig. 6g). The parietal layer of Bowman’s capsule of renal corpuscles and the delicate basement membranes of endothelial cells in the capillary tuft were positive for PAS staining. Additionally, the cytoplasm of the lining epithelial cells of the proximal convoluted tubules, the luminal brush border, and its basement membrane contained positive reaction substances to the PAS stain, indicating the presence of carbohydrate materials. However, PAS-reactivity in the glomerular tufts, brush borders, and basement membranes of the renal tubules of both experimental groups, Sov and Sov + Ri groups (Fig. 6h and i, respectively), was slightly less to somewhat greater than that observed in the control group.

The mercury bromophenol blue stain revealed intense staining for proteins in the cytoplasm, nucleus, and cell membrane of renal cells and glomeruli in sections of all experimental groups: the control, Sov, and Sov + Ri groups (Fig. 6j,k, and l, respectively).

## Discussion

Sofosbuvir aids the body’s immune response against HCV infection by reducing the amount of HCV in the body when combined with other antiviral medications. The treatment regimen involving sofosbuvir and ribavirin for CHC is more efficient than sofosbuvir alone, requires fewer doses, and results in fewer adverse effects^29^. Further studies showed that sofosbuvir and ribavirin combined treatment decreased the risk of HCV infection of the newly transplanted liver in adult patients^30^. This study revealed specific toxicity effects brought on by the simultaneous injection of sofosbuvir and ribavirin. It is yet unclear how these medication responses are caused.

Cell distribution width (RDW %), mean cell hemoglobin (MCH), and mean cell volume (MCV) are parameters routinely evaluated in a complete blood count (CBC), providing valuable information about distribution dispersion. Changes in hematological parameters serve as reliable indicators of medication and substance toxicity. Our study findings revealed that treatment with either sofosbuvir alone or in combination with ribavirin significantly reduced levels of hemoglobin (Hb), packed cell volume (PCV), red blood cells (RBCs), white blood cells (WBCs), including neutrophils, basophils, and platelets. Additionally, both treatments led to a significant decrease in MCH, while MCV showed a non-significant decrease, and RDW% showed a significant increase in the group treated with sofosbuvir alone or in combination with ribavirin. These results suggest the presence of microcytic hypochromic anemia characterized by low MCV and MCH levels. Low MCV and MCH levels are indicative of iron deficiency.

Previous research has indicated that increased extravascular removal through erythrophagocytosis leads to red blood cell (RBC) membrane oxidation, a phenomenon commonly associated with ribavirin-induced anemia^31^. A recent clinical study of 3000 patients with chronic HCV infection treated with sofosbuvir and ribavirin for 24 weeks revealed a significant reduction in hemoglobin concentrations during treatment^32^. Another study confirmed that ribavirin treatment is frequently linked with anemia^33^. Ribavirin’s toxic effects involve inhibiting intracellular energy metabolism and oxidative damage to cell membranes, which accelerates extravascular hemolysis by the reticuloendothelial system^31^. According to research by Ronzoni et al.^34^, peg-IFNa with ribavirin therapy suppresses bone marrow. Additionally, Fellay et al.^35^ demonstrated that the inosine triphosphatase (*ITPA*) gene contains single nucleotide polymorphisms (SNPs) that are related to ribavirin-induced anemia and can be utilized as a marker to indicate the degree of anemia in HCV patients receiving DAAs therapy that includes ribavirin^35^.

Additionally, the ribavirin group and the group treated with sofosbuvir plus ribavirin experienced a significant increase in RDW%. Red blood cell size is a reliable signal for diagnosing erythropoiesis disease in the same scenario. The heterogeneity of peripheral RBCs directly affects RDW%. As a result, the RBC distribution width typically contains varied RBC populations at various phases of maturation and ages.

RDW% might improve our ability to grasp the causes driving anemia development, such as distinct RBC maturation problems or RBC destruction due to hemolysis or bleeding. Variations in RBC size distribution imply variations between RBC production and RBC age^36^. Other investigations agree with the present study, which discovered reduced white blood cells, neutrophils, and monocyte levels while using direct antiviral medications. Low leucocytes were discovered when ribavirin was given as a preventative following liver transplantation. Additionally, administering ribavirin frequently resulted in infections and may be an immune suppressant that worsens viral and bacterial illnesses. The number of monocytes and granulocytes dramatically increased in the group that received sofosbuvir and ribavirin treatment. As is well known, granulocytosis and monocytosis are frequent signs of parasite infection, persistent inflammation, severe depression, and stress. In addition to previous hematological changes, platelet numbers decreased significantly in the sofosbuvir plus ribavirin group, in line with another study using ribavirin as a prophylactic treatment after liver transplantation^37^. Furthermore, previous hematological alterations may have arisen from bone marrow suppression induced by the synergistic effect of sofosbuvir with ribavirin^38,39^. Histopathological observations supported the biochemical data of this study, which indicated a reduction in the number of bone marrow cells.

Compared to the control group, the sofosbuvir and sofosbuvir-ribavirin groups significantly declined the mean total sperm motility percentage. Zowail and Khater’s^40^ study demonstrated that sofosbuvir and ribavirin as antiviral drugs cause cytogenetic impacts, including chromosome ring cancellation, chromatid discontinuity, centromeric weakening, and end-to-end combinations. Moreover, treatment with ribavirin reduces the amount of glutathione in the sperm cells and increases their susceptibility to sperm poisoning and inhibition. There is an inverse relationship between the ratio of glutathione in the cells and the form of spermatozoa. Additionally, changes in lysozyme activity and glutathione levels play a significant role in sperm malformations. Therefore, ribavirin use over an extended period in humans has negative consequences for fertility^41^.

In the Elghazouly and Yassien^42^ study of the fundus of the stomach, the great affinity of sofosbuvir as a nucleotide analog for polymerases of mitochondria led to mitochondrial injury and cell death. They added that damaged mitochondria cause’ oxidative stress by secreting higher levels of reactive oxygen species (ROS), which in turn induce cellular damage in the fundus of the stomach. Recent studies have stated that mitochondrial reactive oxygen species (mROS) act as signaling molecules to stimulate proinflammatory cytokine release through NF_k_B signaling activation. Moreover, the inducible nitric oxide synthase (iNOS) is activated by NF_k_B, producing a significant amount of ROS in the form of nitric oxide (NO). The cytotoxic compound peroxy-nitrite is produced by combining NO with superoxide anions, thereby leading to lipid peroxidation and interstitial fibrosis^42^. Our study suggests that mitochondrial death occurs in the epididymis, which is confirmed by the appearance of collagen in the epidermis, resulting in a significant lowering of the mean total sperm motility percent in the sofosbuvir and sofosbuvir-ribavirin groups. Mitochondria are needed for the energy production of the cells, and the motility of the sperm requires normal mitochondrial function^43^.

Additionally, because these drugs may produce hypopituitarism, either sofosbuvir alone or sofosbuvir with ribavirin has the same impact on the significant lowering of serum T_4_ and serum TSH and MCH and the significant rise of RDW% in CBC^44^ because a deficit of T_4_ may decrease iron absorption and cause hypochromic anemia^45^. Besides, sofosbuvir alone or in combination with ribavirin has the same effect on lowering insulin levels because of a reduction in insulin resistance, insulin plasma concentrations, and an increase in insulin sensitivity, so sofosbuvir has a protective effect on the incidence of diabetes^46^. Our study found an increase in serum cortisol level in the sofosbuvir group only because of hypoglycemia^47^ as a compensatory mechanism^48^.

Moreover, the sofosbuvir or the sofosbuvir-ribavirin group had a significantly lower AFP. Sofosbuvir, or its combination with ribavirin, is associated with a lower risk of developing hepatocellular carcinoma. Moreover, the combination decreases the conflicting interpretation of AFP during the regular follow-up of cirrhosis patients^49^.

In our study, the sofosbuvir-ribavirin group had a significant decrease in serum ALP compared to both groups because ribavirin may decrease serum magnesium and zinc levels due to a deficiency of T_4_^50^. Furthermore, the sofosbuvir-ribavirin group had a significant decrease in serum GOT compared to the sofosbuvir group and a decrease in serum GPT compared with other groups because ribavirin may cause vitamin B_6_ deficiency^51^. The sofosbuvir group had a significant rise in serum GOT levels compared to other groups because sofosbuvir may cause acute pancreatitis^52^. Besides, significantly increased lipase in the sofosbuvir and sofosbuvir-ribavirin groups confirmed sofosbuvir-induced pancreatitis.

Furthermore, the sofosbuvir group had a significant decrease in serum *ADA* and lymphocytes in CBC. This means that sofosbuvir may cause combined immunodeficiency by accumulating toxic purine degradation by-products, most potently affecting lymphocytes as lymphopenia (affecting T cells, B cells, and NK cells), leading to adenosine deaminase deficiency^53^. Mutations in the *ADA* gene are the root cause of adenosine deaminase deficiency. The adenosine deaminase gene contains instructions for making the enzyme. Although this enzyme is present throughout the body, lymphocytes, a specific type of white blood cell, are where it is most active^53^. Moreover, the sofosbuvir-ribavirin group had a significant increase in serum *ADA* because ribavirin may improve sofosbuvir-induced immunodeficiency.

Histopathological analysis of the splenic sections from the sofosbuvir and sofosbuvir-ribavirin groups revealed trabeculae traversing the central part of the splenic section, an increased amount of collagen fibers, markedly thickened trabeculae with an increased amount of collagen fibers, and markedly congested blood vessels, particularly evident in the spleen of the sofosbuvir-ribavirin group. Moreover, there was a slight deposition of collagen fibers observed between the renal tubules, along with an increase in the number of collagen fibers in the interstitial connective tissue surrounding epididymal ducts in both the sofosbuvir and sofosbuvir-ribavirin groups. According to the Elghazouly and Yassien^42^ study, the histopathological findings brought on by sofosbuvir were due to cell death and mitochondrial injury^42^. Alvia et al.^54^ explained these histological results by proposing that ribavirin may have induced an arterial and venous obstruction that led to vascular congestion and edema. Vascular congestion and edema typically co-occur, as congestion of capillaries can lead to edema due to increased fluid leakage. The stagnant deoxygenated blood leads to hypoxia in long-standing overfilling of vessels due to decreased outflow. This can cause parenchymal cell degeneration with the formation of scar tissue^54^.

## Conclusion

Sofosbuvir and ribavirin exhibit a synergistic effect on myelosuppression. Therefore, regular complete blood count (CBC) monitoring is essential during therapy to detect complications promptly. If CBC parameters show signs of impairment, dose reduction or switching to alternative drugs should be considered by the physician. Despite causing iron deficiency anemia, sofosbuvir demonstrates a protective effect against diabetes, a risk that is heightened with ribavirin. The combination of sofosbuvir, with or without ribavirin, is associated with a decreased risk of hepatocellular carcinoma, making it a safe treatment option for HCV-infected patients with diabetes mellitus or hepatic focal lesions. Ribavirin may contribute to T_4_ deficiency via hypopituitarism and vitamin B_6_ deficiency, an issue not observed with sofosbuvir alone. Therefore, thyroid function should be monitored during therapy, and vitamin B_6_ supplementation may mitigate the potential side effects of this combination in HCV infection treatment. Sofosbuvir is linked to acute pancreatitis and combined immunodeficiency. However, ribavirin’s presence enhances the immunodeficiency caused by sofosbuvir, suggesting that combining ribavirin with sofosbuvir mitigates the side effects of both drugs when used alone.

## Data Availability

All data generated or analyzed during this study are included in this published article.
